# Prevalence and risk factors for severe linezolid-associated thrombocytopenia in pediatric patients: An analysis of a public database

**DOI:** 10.1097/MD.0000000000034059

**Published:** 2023-06-16

**Authors:** Shibo Yang, Wencheng Guo, Ming Chen, Jindong Hu, Nana Feng, Mohan Ju, Yiyi Qian

**Affiliations:** a Department of Emergency, Huashan Hospital, Fudan University, Shanghai, People’s Republic of China; b Department of Vascular Surgery, Huashan Hospital, Fudan University, Shanghai, People’s Republic of China; c Department of Respiratory Medicine, Tongde Hospital of Zhejiang Province, Hangzhou, Zhejiang, People’s Republic of China; d Department of Integrative Medicine, Huashan Hospital, Fudan University, Shanghai, People’s Republic of China; e Department of Respiratory and Critical Medicine, Shanghai Eighth People’s Hospital Affiliated to Jiangsu University, Shanghai, People’s Republic of China; f Institute of Antibiotics, Huashan Hospital, Fudan University, Shanghai, People’s Republic of China; g Department of Infectious Diseases, Zhongshan Hospital, Fudan University, Shanghai, People’s Republic of China.

**Keywords:** linezolid, pediatric population, risk factors, severe thrombocytopenia

## Abstract

Linezolid is widely used in various clinical settings. Studies have revealed that it may cause thrombocytopenia in adults. However, the correlation between the use of linezolid and thrombocytopenia in pediatric patients is still unclear. This study aimed to identify the impact of Linezolid on the occurrence of thrombocytopenia in children. A retrospective observational study was conducted using data on patients treated with linezolid from the Pediatric Intensive Care clinical database. Univariate and multiple logistic regression analyses were performed to identify the risk factors of linezolid-related severe thrombocytopenia. A total of 134 patients were included. The prevalence of severe thrombocytopenia was 8.96% (12/134). Univariate analysis indicated that the severe thrombocytopenia group showed significantly higher proportion of concomitant carbapenem (75% vs 44.3%; *P* < .05) and piperacillin/tazobactam (25% vs 6.6%; *P* < .05) than that of the non-severe thrombocytopenia group. Multivariate analysis also revealed that the occurrence of severe thrombocytopenia was significantly associated with concurrent use of carbapenem (odd ratio = 4.058; 95% confidence interval: 1.012–16.274; *P* = .048) and piperacillin/tazobactam (odd ratio = 5.335; 95% confidence interval: 1.117–25.478; *P* = .036). 75% of patients (9/12) developed severe thrombocytopenia within the first 7 days of linezolid use. The concomitant use of carbapenem and piperacillin/tazobactam was associated with an increased probability of severe thrombocytopenia in pediatric patients undergoing linezolid treatment. Further prospective clinical studies are required, and more detailed mechanisms of blood toxicity in pediatric patients must be investigated.

## 1. Introduction

Linezolid is the first member of the oxazolidinone family and is widely used in clinical settings because of its potent antimicrobial activity against methicillin-resistant *Staphylococcus aureus*, penicillin-resistant *Streptococcus pneumoniae*, vancomycin-resistant *Enterococcus*, and other multidrug-resistant gram-positive bacteria.^[1]^ Linezolid exerts its antibacterial activity by binding to the 23S site of ribosomal RNA on the 50S subunit of bacteria, inhibiting 70S complex formation and bacterial protein synthesis.^[2]^ The absorption of linezolid is both rapid and complete after oral administration, resulting in a 100% absolute bioavailability rate. Thus, there is no requirement to adjust the dosage of Linezolid when switching between oral and intravenous administration.^[3]^ This characteristic supports the practice of sequential oral administration of linezolid following initial intravenous administration. The plasma protein binding rate of linezolid was found to be approximately 31% and was not concentration-dependent. In healthy volunteers, the distribution volume of linezolid in stable state was 40 to 50L on average, which is close to the total body fluid. Both animal and human studies have demonstrated that linezolid has strong tissue penetration and can be rapidly distributed in well-perfused tissues.^[4,5]^ Linezolid is associated with several common adverse effects including diarrhea, headache, nausea, vomiting, and insomnia. Laboratory tests may reveal a decreased platelet count, decreased White blood cell count, or abnormal liver function. Patients undergoing longer treatment courses may face an increased risk of developing peripheral neuropathy and optic neuropathy.^[6]^ It is important to note that Linezolid is a mildly reversible, nonselective monoamine oxidase inhibitor that can interact with epinephrine or serotonin drugs to produce symptoms of serotonin syndrome, such as fever and elevated blood pressure.^[7]^ Nowadays, thrombocytopenia caused by the drug has attracted increasing attention. A few studies have reported some risk factors in patients treated with linezolid for thrombocytopenia, such as poorer renal function, longer treatment duration, and the coexisting condition of malignancy.^[8–10]^ Most studies conducted to date concerning this issue have always focused on adult populations, with only a few demonstrating the impact of linezolid use on the development of thrombocytopenia in pediatric patients.^[11,12]^ Therefore, we conducted this study to investigate the relationship between linezolid use and severe thrombocytopenia and identify the potential risk factors for severe linezolid-associated thrombocytopenia in pediatric patients.

## 2. Methods

### 2.1. Data collection

This retrospective observational study was conducted according to the Declaration of Helsinki (as revised in 2013) using data from the Pediatric Intensive Care database (PIC database, version 1.1). This is a publicly accessible single-center database containing the clinical information of approximately 13,000 patients admitted to the Children Hospital of Zhejiang University (Hangzhou, Zhejiang, China) from 2010 to 2018.^[13]^ The following data of each patient were extracted using PostgreSQL (Version 9.6) and R language (Version 3.6.0) by 2 independent researchers: demographic characteristics, diagnosis at admission/discharge, dates and results of laboratory tests and prescriptions, and other related data. Baseline laboratory tests were defined as the latest test taken within 72 hours before the start of treatment. Concomitant drugs were defined as medications administered for an overlapping duration of more than 48 hours with linezolid treatment.

### 2.2. Patient selection

Individuals aged between 28 days and 18 years are defined as pediatric patients in our study. All pediatric patients treated with linezolid for 5 days or longer were included. The exclusion criteria were patients without baseline platelet counts, patients with baseline platelet counts of <100 × 10^9^/L, patients diagnosed with hemato-oncologic diseases and bleeding disorders, and patients without monitoring of platelet counts (at least every 48 hours) during linezolid therapy. Severe thrombocytopenia was defined as a reduction in platelet counts of >75% from baseline or <50 × 10^9^/L. The study protocol was approved by the Institutional Review Board of the Children Hospital, Zhejiang University School of Medicine (Hangzhou, China). The requirement for obtaining individual patient consent was waived as the study did not have an impact on clinical care and all protected health information was de-identified.

### 2.3. Statistical analysis

Data were processed with SPSS for Windows (Version 22.0). Continuous variables were analyzed using the Student *t* test or Mann–Whitney U test, whereas categorical variables were analyzed using the chi-squared test or Fisher exact test. Variables with *P* value < .1 in univariate analysis were analyzed using logistic regression. Statistical significance was set at *P* < .05. Missing variables were addressed with the multiple imputation technique.

## 3. Results

### 3.1. Patient cohort

**Figure 1.**
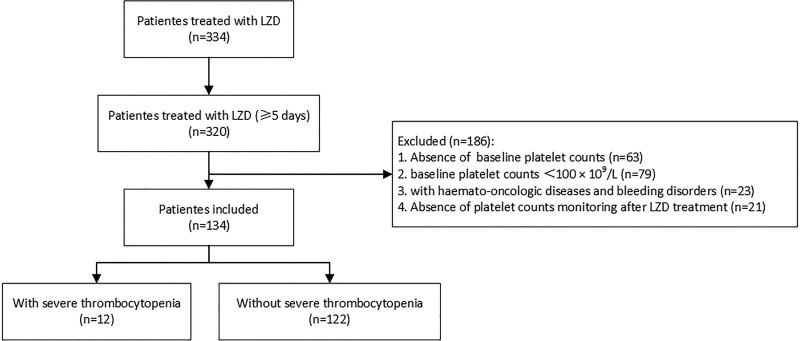
Flow chart of patients selection. LZD = linezolid.

Of the patients in the PIC database, 320 received linezolid treatment for 5 days or longer. Among them, 186 patients were excluded based on the exclusion criteria. The remaining 134 patients were included for further analysis (Fig. 1).

### 3.2. Demographics and clinical characteristics

Table 1 shows the baseline characteristics of the 134 patients. In total, 49.3% (66/134) of patients underwent linezolid treatment from 8 to 14 days, with the median duration being 10 days (inter quartile range: 7–13 days). Respiratory tract diseases were the most common comorbidity and were found in 29.9% (40/134) of patients. A total of 47 (35.1%) patients were treated according to the culture result, whereas 87 (64.9%) patients were empirically treated. The predominantly identified pathogens were Methicillin-resistant Coagulase-negative *staphylococci* (in 17 patients) and Streptococcus spp. (in 15 patients). MRSA was recovered in 5 patients (3.7%). During linezolid therapy, carbapenem, piperacillin/tazobactam, and sulfamethoxazole-trimethoprim were concomitantly used in 47.01%, 8.21%, and 3.73% of patients, respectively.

**Table 1:** Characteristics of enrolled patients.

Features	n (%)
Total	134
Severe thrombocytopenia	12 (9.0%)
Age	
<3 yr	83 (61.9%)
3–18 yr	51 (38.1%)
Sex (female)	41 (30.6%)
Treatment duration	
5–7 d	42 (31.3%)
8–14 d	66 (49.3%)
>14 d	26 (19.4%)
Comorbidities	
Respiratory tract diseases	40 (29.9%)
Heart diseases	31 (23.1%)
Central nervous system diseases	21 (15.7%)
Autoimmune diseases	8 (6.0%)
Digestive system disease	13 (9.7%)
Urinary system disease	2 (1.5%)
Musculoskeletal system disease	6 (4.5%)
Endocrine system disease	4 (3.0%)
Skin and soft-tissue disease	6 (4.5%)
Inherited metabolic disorders	3 (2.2%)
Solid organ malignancy	9 (6.7%)
Sepsis	16 (12.0%)
Isolated microorganisms	
Enterococci	10 (7.5%)
MRCoNS	17 (12.7%)
MSCoNS	4 (3.0%)
Streptococcus spp.	15 (11.2%)
MRSA	5 (3.7%)
MSSA	4 (3.0%)
Concomitant drugs	
Carbapenem	63 (47.0%)
Piperacillin-tazobactam	11 (8.2%)
SMZ-TMP	5 (3.7%)
Valproic acid	10 (7.5%)
Heparin/LMWH	73 (54.5%)
ICU admission	57 (42.5%)
In-hospital mortality	7 (5.2%)

ICU = intensive care unit, LMWH = low-molecular-weight heparin, MRCoNS = methicillin-resistant coagulase-negative Staphylococcus, MRSA = methicillin-resistant *Staphylococcus aureus*, MSCoNS = methicillin- sensitive coagulase-negative Staphylococcus, MSSA = methicillin-sensitive *Staphylococcus aureus*, SMZ-TMP = sulfamethoxazole-trimethoprim.

### 3.3. Linezolid use and development of severe thrombocytopenia

During linezolid treatment, 12 patients (8.96%) developed severe thrombocytopenia (Table 2). The risk factors associated with thrombocytopenia were investigated using univariate analysis between 2 groups (Table 3). In the severe thrombocytopenia group, the concomitant use of carbapenem and piperacillin/tazobactam was significantly higher than that in the non-severe thrombocytopenia group (75% vs 44.3%, *P* < .05; 25% vs 6.6%, *P* < .05; respectively). There was no significant difference between the 2 groups in linezolid treatment duration, baseline laboratory data (including hemoglobin counts, platelet counts, and serum creatinine level), and use of other concomitant drugs (including sulfamethoxazole-trimethoprim, valproic acid, and heparin/low molecular weight heparin). Further multivariate analysis also revealed that the concomitant use of carbapenem (odd ratio = 4.058; 95% confidence interval: 1.012–16.274; *P* = .048) and piperacillin/tazobactam (odd ratio = 5.335; 95% confidence interval: 1.117–25.478; *P* = .036) with linezolid was a risk factor for the development of severe linezolid-associated thrombocytopenia.

**Table 2:** Characteristics of patients with severe thrombocytopenia.

Pat. No	Age	Sex	Baseline PLT†	Treatment duration (d)	PLT after treatment†	Concomitant treatment	Comorbidity
1	5.9 yr	F	128	18	43	SMZ-TMP Carbapenem	Sepsis
2	6.9 yr	M	120	10	20	Carbapenem	Sepsis Respiratory tract diseases
3	68 d	M	300	9	47	Carbapenem	Respiratory tract diseases
4	79 d	F	487	20	41	Carbapenem Heparin/LMWH	Respiratory tract diseases
5	8.0 yr	F	128	6	6	Carbapenem	Sepsis
6	209 d	M	158	11	38	Piperacillin-tazobactam	Endocrine system disease
7	160 d	M	390	6	37	Carbapenem	Respiratory tract diseases Digestive system disease
8	3.7 yr	M	308	8	4		Central nervous system diseases
9	31 d	F	113	5	46	Carbapenem Heparin/LMWH	Heart diseases
10	49 d	M	440	13	13	Carbapenem Heparin/LMWH	Digestive system disease
11	33 d	F	158	7	49	Carbapenem Heparin/LMWH	Respiratory tract diseases Heart diseases
12	84 d	F	354	14	63		Respiratory tract diseases

d = days, F = female, LMWH = low-molecular-weight heparin, M = male, PLT = platelet, SMZ-TMP = sulfamethoxazole-trimethoprim, yr = years.

† Counted as ×10^9^/L.

**Table 3:** Univariate analysis of risk factors for severe thrombocytopenia.

	Severe thrombocytopenia (n = 12)	Non-severe thrombocytopenia (n = 122)	*P* value
Age, n (%)			
<3 yr	8 (66.7%)	75 (61.5%)	.967
3–18 yr	4 (33.3%)	47 (38.5%)	
Sex (female), n (%)	5 (41.7%)	36 (29.5%)	.383
Treatment duration (d), median (IQR)	9.5 (6.8–13.3)	10 (7–13)	.737
Treatment duration > 14 d, n (%)	2 (16.7%)	24 (19.7%)	.802
Dose of Linezolid (mg/d), mean ± SD	600.9 ± 289.4	582.7 ± 298.0	.463
Comorbidities, n (%)			
Respiratory tract diseases	6 (50%)	34 (27.9%)	.11
Heart diseases	2 (16.7%)	29 (23.8%)	.578
Central nervous system diseases	1 (8.3%)	20 (16.4%)	.464
Autoimmune diseases	0 (0%)	8 (6.6%)	.36
Digestive system disease	2 (16.7%)	11 (9.0%)	.393
Urinary system disease	0 (0%)	6 (4.9%)	.432
Musculoskeletal system disease	0 (0%)	2 (1.6%)	.655
Endocrine system disease	1 (8.3%)	3 (2.5%)	.254
Skin and soft-tissue disease	0 (0%)	6 (4.9%)	.432
Inherited metabolic disorders	0 (0%)	3 (2.5%)	.583
Solid organ malignancy	0 (0%)	9 (7.4%)	.33
Sepsis	3 (25%)	13 (10.7%)	.144
Baseline laboratory results			
White blood cell(*10^9^/L), median (IQR)	14.9 (7.5–25.7)	12.9 (8.5–18.7)	.637
Red blood cell (*10^12^/L), mean ± SD	3.7 ± 0.8	3.8 ± 0.7	.624
Hemoglobin (g/L), mean ± SD	104.8 ± 19.6	106 ± 20.3	.849
Platelet counts (*10^9^/L), median (IQR)	229 (128–363)	301 (210.3-411.8)	.13
Serum creatinine (µmol/L), median (IQR)†	40.5 (29–52.3)	41.85 (33–60)	.648
Concomitant drugs			
Carbapenem, n (%)	9 (75%)	54 (44.3%)	.042
Piperacillin-tazobactam, n (%)	3 (25%)	8 (6.6%)	.026
SMZ-TMP, n (%)	1 (8.3%)	4 (3.3%)	.378
Valproic acid, n (%)	0 (0%)	10 (8.2%)	.303
Heparin/LMWH, n (%)	4 (33.3%)	69 (56.6%)	.123
ICU admission, n (%)	6 (50%)	51 (41.8%)	.584

ICU = intensive care unit, IQR = interquartile range, LMWH = low-molecular-weight heparin, SD = standard deviation, SMZ-TMP = sulfamethoxazole-trimethoprim.

† Missing data of 10 patients for baseline serum creatinine was addressed with the multiple imputation technique.

### 3.4. Time of onset of severe thrombocytopenia

**Figure 2.**
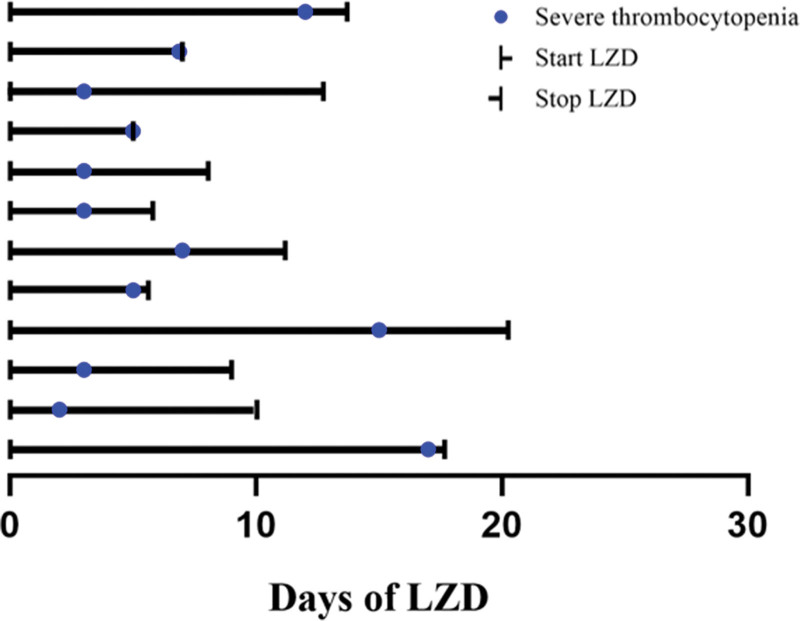
Timeline of the 12 patients with LZD induced severe thrombocytopenia. LZD = linezolid.

Figure 2 shows the timelines of the 12 patients who developed severe thrombocytopenia, of whom 9 patients (75%) developed severe thrombocytopenia within the first 7 days of linezolid use. The median time of onset of severe thrombocytopenia was 5 days (inter quartile range: 3–8.25 days).

## 4. Discussion

To our knowledge, this is the first study to investigate the prevalence and risk factors of linezolid-associated severe thrombocytopenia in pediatric patients in China. Results showed that 8.96% of patients receiving linezolid treatment developed severe thrombocytopenia, and the concomitant use of carbapenem and piperacillin/tazobactam may elevate the risk of this adverse event.

Previous studies have indicated that linezolid-associated hematologic adverse events often occurred at least 14 to 21 days after the start of treatment.^[14–16]^ Prolonged treatment duration increases the probability of hematologic adverse events caused by linezolid. A recent meta-analysis showed that the incidence of thrombocytopenia increased by 5 times in pediatric patients receiving linezolid treatment for more than 14 days.^[17]^ In the present study, we could not identify long-term treatment as a risk factor for linezolid-associated severe thrombocytopenia. However, most cases of severe thrombocytopenia occurred within the first 10 days of linezolid treatment. Hence, severe thrombocytopenia may occur earlier than we expected in the course of antimicrobial therapy, and careful dynamic monitoring of platelet counts might be essential.

We also analyzed the use of concomitant drugs, including several antibiotics, antiseizure medications (valproic acid), heparin, and low-molecular-weight heparin that may induce thrombocytopenia. Results showed that the concomitant use of carbapenem and piperacillin/tazobactam with linezolid was a risk factor for the development of severe thrombocytopenia in pediatric patients. This finding was similar to that reported by Chen et al^[16]^ and Kiliç et al^[18]^ who found that adult patients undergoing carbapenem–linezolid combination therapy had higher risk of developing thrombocytopenia. It must be noted that the concomitant use of linezolid and carbapenem or piperacillin/tazobactam provides broad antibacterial spectrum and is widely applied in clinical settings, especially for patients with severe conditions or as empiric treatment. Therefore, it is necessary to focus more on correlating the adverse event of thrombocytopenia of this regimen and the potential mechanism. Interestingly, carbapenem and piperacillin/tazobactam, both of which are from the group of β-lactam antimicrobials, are rarely considered as the cause of thrombocytopenia. There is limited literature on thrombocytopenia induced by carbapenem or piperacillin/tazobactam.^[19–21]^ The mechanism by which the combination therapy increases the risk is unclear. The possible explanations include the additional myelosuppression effect that inhibits the production of platelets and the accelerated activation of the complement system to promote the destruction of platelets.^[22–24]^ However, further studies are necessary.

We also analyzed other potential risk factors associated with linezolid-induced thrombocytopenia, including renal function and baseline platelet counts, but we found no correlation. However, Hanai et al^[14]^ reported increased risk of developing linezolid-associated thrombocytopenia in adult patients when their creatinine clearance was <60 mL/min. In pediatric patients, Johns et al^[11]^ found that renal impairment before the start of linezolid treatment was an independent risk factor for thrombocytopenia. Although linezolid is eliminated from the body primarily through nonrenal pathways, elevated drug concentrations can be found in patients with renal insufficiency, increasing the probability of thrombocytopenia. Our failure to identify the correlation between renal function and severe thrombocytopenia may be explained by the fact that we could use only baseline serum creatinine as the index of patients’ renal function, instead of estimated glomerular filtration rate or creatinine clearance, due to missing data in the database, such as height and weight. We also found no correlation between drug dosage and decline in platelet count, since the dose of linezolid must be adjusted according to the weight of patient in populations younger than 12 years old, the absence of complete data may lead to a bias in the results.

A few studies have revealed the relationship between baseline platelet counts and thrombocytopenia development. For instance, Choi et al^[25]^ reported that a baseline platelet counts of <150 × 10^9^/L was associated with thrombocytopenia occurrence, whereas Lima et al identified a baseline platelet counts of <200 × 10^9^/L.^[26]^ Our failure to detect a significant correlation may be attributed to the relatively small simple size.

Compared to vancomycin, linezolid has the advantage of oral administration and a higher bioavailability, making it a commonly utilized option in sequential treatment regimens for long-term management of infectious diseases. In clinical practice, there have been instances where patients continue to take linezolid orally after they leave the hospital. This presents a significant challenge and emphasizes the need for adequate patient education regarding potential adverse drug reactions and the conditions that may augment their likelihood. Proactive monitoring of patient outcomes through methods such as telephonic follow-up or establishment of a support group is imperative to detect any adverse events in a timely manner. In case of severe adverse reactions, such as severe thrombocytopenia, prompt notification of the patient family and initiation of appropriate medical intervention is crucial.

There were inherent limitations in this study. First, as mentioned earlier, the inaccessibility of certain information in the PIC database, such as height, weight, and serum drug concentration, may negatively affect our conclusion. Second, the retrospective nature of this single-center study and the small sample size may restrict the generalization of our result.

## 5. Conclusion

The concomitant use of carbapenem and piperacillin/tazobactam was associated with an increased probability of severe thrombocytopenia in pediatric patients undergoing linezolid treatment. However, due to the small sample size and missing data of clinical information, further prospective clinical studies are required, and more detailed mechanisms of blood toxicity in pediatric patients must be investigated.

## Acknowledgments

We would like to thank Prof Minhao Li, the PIC database manager, for the grant of access to the database as well as its tutorial of use. We also appreciate Miss. Mengzhi Zheng for her technical support to this study. We would like to thank professional editors at Goldediting for their help in polishing our paper.

## Author contributions

**Conceptualization:** Mohan Ju, Yiyi Qian.

**Data curation:** Shibo Yang, Wencheng Guo.

**Formal analysis:** Shibo Yang.

**Funding acquisition:** Mohan Ju, Ming Chen, Nana Feng.

**Investigation:** Wencheng Guo, Jindong Hu.

**Methodology:** Mohan Ju, Yiyi Qian, Wencheng Guo.

**Software:** Wencheng Guo.

**Supervision:** Ming Chen, Nana Feng.

**Validation:** Nana Feng.

**Visualization:** Nana Feng.

**Writing – original draft:** Mohan Ju, Jindong Hu.

**Writing – review & editing:** Mohan Ju.
